# Aldose Reductase Inhibitor Protects against Hyperglycemic Stress by Activating Nrf2-Dependent Antioxidant Proteins

**DOI:** 10.1155/2017/6785852

**Published:** 2017-06-27

**Authors:** Kirtikar Shukla, Pabitra Bikash Pal, Himangshu Sonowal, Satish K. Srivastava, Kota V. Ramana

**Affiliations:** Department of Biochemistry and Molecular Biology, University of Texas Medical Branch, Galveston, TX 77555, USA

## Abstract

We have shown earlier that pretreatment of cultured cells with aldose reductase (AR) inhibitors prevents hyperglycemia-induced mitogenic and proinflammatory responses. However, the effects of AR inhibitors on Nrf2-mediated anti-inflammatory responses have not been elucidated yet. We have investigated how AR inhibitor fidarestat protects high glucose- (HG-) induced cell viability changes by increasing the expression of Nrf2 and its dependent phase II antioxidant enzymes. Fidarestat pretreatment prevents HG (25 mM)-induced Thp1 monocyte viability. Further, treatment of Thp1 monocytes with fidarestat caused a time-dependent increase in the expression as well as the DNA-binding activity of Nrf2. In addition, fidarestat augmented the HG-induced Nrf2 expression and activity and also upregulated the expression of Nrf2-dependent proteins such as hemeoxygenase-1 (HO1) and NQO1 in Thp1 cells. Similarly, treatment with AR inhibitor also induced the expression of Nrf2 and HO1 in STZ-induced diabetic mice heart and kidney tissues. Further, AR inhibition increased the HG-induced expression of antioxidant enzymes such as SOD and catalase and activation of AMPK-*α*1 in Thp1 cells. Our results thus suggest that pretreatment with AR inhibitor prepares the monocytes against hyperglycemic stress by overexpressing the Nrf2-dependent antioxidative proteins.

## 1. Introduction

Hyperglycemia is a major contributor to inflammation, apoptosis, profound vasodilation, tissue damage, and dysfunction in patients with diabetes mellitus [1]. The cytotoxicity of hyperglycemia is mediated by the increase in reactive oxygen species (ROS) which activate NF-*κ*B and AP1 that results in the transcription of inflammatory cytokines [2]. Our recent studies indicate that inhibition of the polyol pathway enzyme aldose reductase (AR) prevents cytokine- and hyperglycemia-induced increase in inflammatory markers in macrophages, vascular cells, and diabetic mice [3, 4] by preventing the activation of NF-*κ*B- and AP1-induced proinflammatory signals [3, 5]. We have shown that preincubation with AR inhibitor prevents hyperglycemia- and cytokine-induced proliferation of vascular cells and apoptosis of macrophages [6–8]. While these studies indicate that inhibition of AR could prevent oxidative stress-induced inflammatory response, the mechanism(s) by which inhibition of AR prepares the cells against oxidative stress is not known.

Previous studies indicate that transcription factor nuclear factor-erythroid-2-related factor 2 (Nrf2) regulates a battery of cytoprotective genes which maintain cellular redox homeostasis. Nrf2 binds to the antioxidant response element (ARE) and transcriptionally regulates the gene expression of several antioxidant and phase II detoxifying enzymes including hemeoxygenase 1 (HO1), NAD(P)H-quinone dehydrogenase 1 (NQO1), *γ*-glutamylcysteine synthetase (GCS), glutathione *S*-transferases (GSTs), and AR [9]. Generally, under nonstress conditions, Nrf2 complexes with an adaptor protein, Keap1, which is a regulator of the proteasomal degradation of Nrf2. However, under stress conditions, Nrf2 dissociates from Keap1 and translocates to the nucleus and transcribes the genes responsible for defense against stress. Thus, it has been well established that activation of the Nrf2 pathway in response to oxidative stress protects the cells and tissues from oxidative injury [10].

Although our previous studies indicate that AR inhibition prevents hyperglycemia-induced NF-*κ*B-dependent inflammatory signals, it is not known how AR inhibition increases the resistance of cells to withstand oxidative stress stimuli initiated by hyperglycemia. Therefore, in this study, we examined our hypothesis that AR inhibition promotes the activation of Nrf2-mediated cytoprotective pathways that protect cells against hyperglycemic stress. Our results suggest that AR inhibitor fidarestat increases the expression as well as the DNA-binding activity of Nrf2 in Thp1 monocytes. In addition, fidarestat increased the expression of Nrf2 downstream target proteins such as HO1 and NQO1 in Thp1 cells and heart and kidney tissues of STZ-induced diabetic mice. AR inhibitor also prevented the expression of antioxidant enzymes such as SOD and catalase. Collectively, our results demonstrate that AR inhibition protects the cells against hyperglycemia-induced changes in the cell viability by activating Nrf2/HO1-mediated antioxidative pathway, which could also account for the anti-inflammatory effects of AR inhibitors in diabetes.

## 2. Materials and Methods

### 2.1. Materials

RPMI-1640, penicillin/streptomycin, and fetal bovine serum (FBS) were purchased from Invitrogen. Antibodies against Nrf2 were obtained from Santa Cruz. Antibodies against KEAP1, HO1, NQO1, phospho-AMPK-*α*1, AMPK-*α*1, GAPDH, and histone H3 were purchased from Cell Signal Inc. Fidarestat was obtained from Livwel Therapeutics Inc., USA. 3-(4,5-dimethylthiazol-2-yl)-2,5-diphenyltetrazolium-bromide (MTT) and other reagents used in Western blot analysis were obtained from Sigma. HO1 assay kit was purchased from Abcam. Nrf2 transcription factor binding assay and superoxide dismutase activity assay kits were purchased from Cayman Chemicals. Catalase activity was determined spectrophotometrically using hydrogen peroxide (30%) (Sigma-Aldrich).

### 2.2. Cell Culture and Treatment

Human Thp1 monocytic cells were obtained from the American Type Culture Collection (ATCC) and cultured in RPMI-1640 medium supplemented with 10% FBS and penicillin/streptomycin at 37°C in a humidified atmosphere of 5% CO_2_. Prior to treatment, cells were serum starved in the respective medium containing 0.1% FBS ± fidarestat (10 *μ*M) for 14 h and further stimulated with high glucose (25 mM; 19.5 mM glucose was added to 5.5 mM glucose containing media) for different time intervals.

### 2.3. Cell Viability

Cell viability was determined using a standard MTT assay [11]. Briefly, Thp1 cells were growth arrested in 0.1% FBS containing RPMI medium. Cells were preincubated with fidarestat (10 *μ*M) for overnight (14 h) at37°C followed by incubation with HG (25 mM) for another 48 h. At the end of incubation period, cells were incubated with 10 *μ*l MTT reagent (5 mg/ml) for 4 h at 37°C. The formazan crystals formed by the viable cells were solubilized by the addition of 100 *μ*l DMSO. Absorbance was measured at 570 nm using a microplate reader. Cell viability was also examined by counting the live and dead cells using a hemocytometer [6]. AR activity (mU/mg protein) was measured spectrophotometrically using glyceraldehyde as a substrate [6]. Nrf2 DNA binding activity was determined by Nrf2 transcription factor assay kit as per the manufacturer's instructions (Cayman Chemical).

### 2.4. Immunoblot Analysis

Nuclear and cytoplasmic proteins from the treated cells were isolated using a nuclear extraction kit (Cayman Chemicals). Protein concentration in the extracts was measured with Bradford reagent (Bio-Rad). Equal amount of proteins were subjected to 12% SDS-PAGE electrophoresis followed by Western blot analysis using specific antibodies against Nrf2, Keap1, HO1, NQO1, AMPK, histone H3, and GAPDH. The antigen-antibody complexes were detected by enhanced Super Signal West Pico Chemiluminescent Substrate (Thermo Scientific). Membranes were stripped with Restore TM PLUS stripping buffer (Thermo Scientific) and used for reprobing with other antibodies or loading controls.

### 2.5. Ablation of Nrf2 by siRNA

Thp1 cells cultured in RPMI 1640 medium containing 10% FBS were incubated with Nrf2-siRNA (120 nM) or siRNA negative control with HiPerFect Transfection Reagent as per the manufacturer's instructions (Qiagen, USA). The cells were incubated in a humidified CO_2_ incubator for 48 h at 37°C. Silencing of Nrf2 was determined by Western blotting.

### 2.6. Quantitative RT-PCR Analysis of *HO1* mRNA

Total RNA was isolated from the treated cells using TRIzol reagent and was quantified by using a nanodrop spectrophotometer (NanoDrop Technologies). TaqMan reverse transcription reagents kit was used for the synthesis of cDNA from total RNA (Life Technologies). Q-PCR amplifications (performed in triplicate) were performed by using 1 *μ*l of cDNA using the iTaq Universal SYBR Green Supermix (Bio-Rad). Housekeeping gene *GAPDH* was used as a normalizer. ABI Prism 7500 Sequence detection system using forward: 5′-CGGGCCAGCAACAAAGTG-3′, and reverse: 5′-CCAGAAAGCTGAGTGTAAGGACC-3′ was used for qPCR analysis of *HO1* gene.

### 2.7. Determination of HO1 and Nrf2 in STZ-Induced Diabetic Mice

Seven-week-old C57BL/6 male mice were purchased from Envigo. Diabetes was induced in mice by injecting a single dose of streptozotocin (STZ; 165 mg/kg, i.p.) and blood glucose levels were measured by a glucometer (True Metrix). The mice with blood glucose levels >400 mg/dl were selected and randomly divided into diabetic and diabetic + fidarestat groups. Fidarestat (10 mg/kg/day, i.p.) was administered to diabetic mice, and the animals were euthanized on day 3.

### 2.8. Statistical Analysis

Data are presented as mean ± SD. The *p* values were determined using the unpaired Student's *t*-test (GraphPad Prism software) and a *p* value of <0.05 considered as statistically significant.

## 3. Results

### 3.1. AR Inhibition Prevents HG-Induced Thp1 Cells Viability

**Figure 1:**
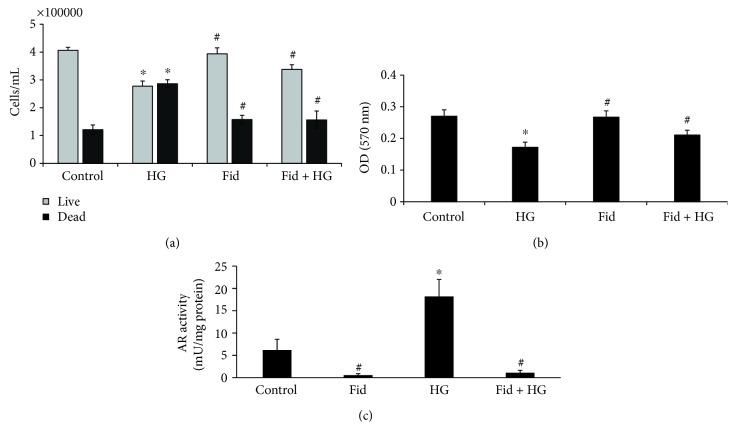
AR inhibition prevents HG-induced Thp1 cell viability. Thp1 cells (3000 cells/well) were pretreated with fidarestat for overnight followed by incubation with HG (25 mM) for another 48 h. (a) Cell viability was determined by MTT assay. (b) Live and dead cell counts were determined by staining with trypan blue using a hemocytometer. (c) AR activity was determined spectrophotometrically using glyceraldehyde as a substrate. Data represent mean ± SD (*n* = 5). ^∗^*p* < 0.01 when compared with control, and #*p* < 0.05 when compared with the HG-treated group.

The effect of AR inhibition on HG-induced Thp1 cells viability was examined by measuring the live and dead cell counts as well as MTT absorbance. The data shown in the Figure 1(a) indicates that HG treatment of Thp1 cells decreased the number of live cells and increased the number of dead cells indicating that HG decreases Thp1 cell viability. However, pretreatment of Thp1 cells with AR inhibitor prevented the HG-induced decrease in the Thp1 cell viability. Similar results were observed when we measured the cell viability by MTT assay (Figure 1(b)). The data shown in Figure 1(c) also indicates that AR activity was significantly increased in the HG-treated Thp1 cells and fidarestat prevented it. These results thus suggest that AR inhibition prevents HG-induced decrease in the cell viability of Thp1 cells.

### 3.2. AR Inhibitor Increases the Expression of Nrf2

**Figure 2:**
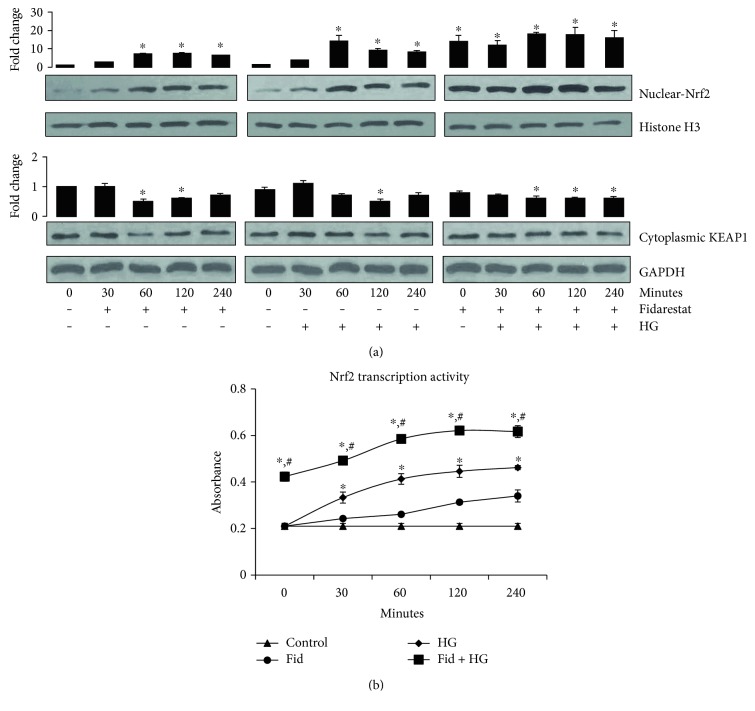
AR inhibition augments HG- induced Nrf2 activation in Thp1 cells. Thp1 cells were treated with fidarestat (10 *μ*M) for indicated times. Subsequently, the cells were also pretreated with fidarestat for overnight followed by incubation with HG for 30, 60, 120, and 240 minutes. Equal amounts of nuclear and cytosolic proteins were subjected to Western blot analysis for the expression of Nrf2 and Keap1, respectively. Histone H3 and GAPDH served as loading controls for nuclear and cytosolic protein extract, respectively. A representative blot from three independent analyses is shown (a). Nrf2 transcription factor assay using the nuclear protein of treated Thp1 cells was carried out using an ELISA kit (b). Data represent mean ± SD (*n* = 5). ^∗^*p* < 0.05 when compared with control, and #*p* < 0.05 when compared with the HG-treated group.

To examine how pretreatment of cells with AR inhibitor prevents HG-induced decrease in Thp1 cell viability, we examined the effect of AR inhibitor on the expression of Nrf2. Pretreatment of Thp1 cells with fidarestat alone or HG alone induced Nrf2 expression in a time-dependent manner. Further, preincubation of cells with fidarestat followed by incubation with HG significantly augmented the HG-induced increase in the expression of Nrf2 (Figure 2(a)). Similarly, treatment of Thp1 cells with HG decreased the expression of Keap1, a negative regulator of Nrf2 and preincubation with fidarestat, followed by HG decreased the expression of the Keap1 protein (Figure 2(a)). We next examined the effect of AR inhibitor on Nrf2 DNA binding activity in Thp1 cells. Nrf2 transcriptional activity increased in the fidarestat-treated Thp1 cells in a time-dependent manner as compared to that in control cells (Figure 2(b)). Further, fidarestat augmented the HG-induced Nrf2 transcriptional activity in Thp1 cells. These results thus suggest that preincubation of cells with AR inhibitor prepares the cells against oxidative insult by inducing the expression of Nrf2.

### 3.3. AR Inhibition Increases the Antioxidative Protein Expressions in Thp1 Cells

**Figure 3:**
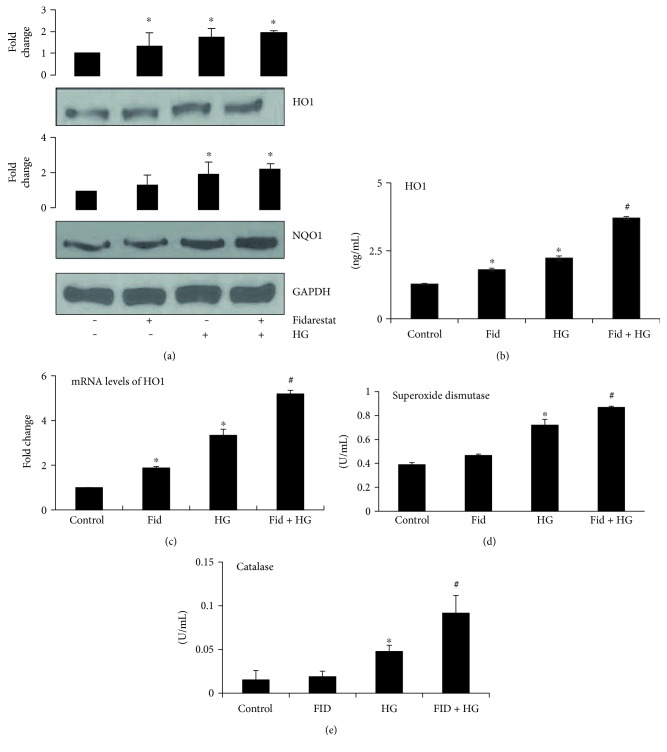
AR inhibitor induces the expression of Nrf2-dependent antioxidant enzymes in HG-treated Thp1 cells. Thp1 cells were pretreated with fidarestat for 14 h followed by incubation with HG (25 mM) for another 24 h. Equal amounts of proteins were subjected to Western blot analysis for the expression of HO1 and NQO1. Fold changes were determined after normalizing with loading control GAPDH. A representative blot from three independent analyses is shown (a). The HO1 levels in the cell lysates were determined by ELISA (b). The mRNA levels of the *HO1* gene in Thp1 cells were determined by RT-PCR as described in Section 2 (c). SOD and catalase activities were analyzed in Thp1 cell lysates by using specific kits as per the manufacturer's instructions (d and e). Data represent mean ± SD (*n* = 5). ^∗^*p* < 0.01 versus control; #*p* < 0.05 when compared with the HG-treated group or the Fidarestat alone-treated group.

We next examined the effect of AR inhibitor on the expression of various Nrf2-dependent antioxidative proteins. Results shown in Figure 3(a) indicate that fidarestat alone or HG alone increased the levels of antioxidant proteins such as HO1 and NQO1 in Thp1 cells. Further, pretreatment with fidarestat followed by HG synergistically increased the HO1 and NQO1 protein expressions in Thp1 cells (Figure 3(a)). Similarly, AR inhibitor also increased the levels of HO1 in Thp1 cell lysates (Figure 3(b)). Furthermore, mRNA expression of *HO1* increased significantly in cells treated with HG in the presence of fidarestat as compared to HG- or fidarestat-treated cells (Figure 3(c)). Furthermore, AR inhibitor also significantly increased the HG-induced SOD as well as catalase activities in Thp1 cells (Figures 3(d) and 3(e)). Thus, our results indicate that pretreatment of Thp1 cells with fidarestat enhances the antioxidant status of the cells as a defense against hyperglycemic stress.

### 3.4. Effect of AR Inhibitor on HG-Induced Cell Viability in Nrf-2-Knockdown Thp1 Cells

**Figure 4:**
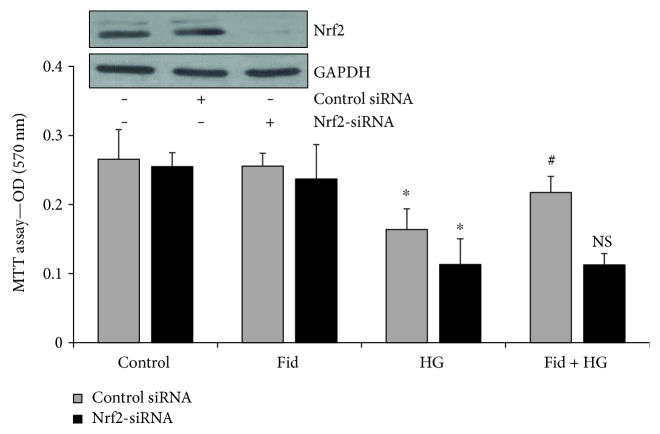
AR-inhibition regulates cell viability of Thp1 cells via Nrf2 activation. The control-siRNA and Nrf2-siRNA-transfected Thp1 cells were treated with HG ± fidarestat for 48 h, and the cell viability was determined by MTT assay. Data represent in mean ± SD (*n* = 6). ^∗^*p* < 0.01 versus control; #*p* < 0.01 versus HG; NS = nonsignificant versus HG-treated cells. The inset shows Western blots for Nrf2 in control and siRNA-transfected cells. GAPDH = loading control.

To examine the effect of Nrf2 on AR-regulated cell growth, we determined the Thp1 cell viability in the Nrf2-siRNA knockdown cells in the absence and presence of fidarestat. Incubation of Nrf2 knockdown cells with HG (25 mM) significantly increased cell death of Thp1 cells when compared to that of control cells (Figure 4). Further, fidarestat prevented the HG-induced Thp1 cell death in control siRNA-transfected cells but not in the Nrf2-siRNA-transfected cells, suggesting that fidarestat prevents Thp1 cell viability by increasing the Nrf2 expression.

### 3.5. AR Inhibitor Increases the Expression of Nrf2 and HO1 in Mouse Diabetic Heart and Kidney Tissues

**Figure 5:**
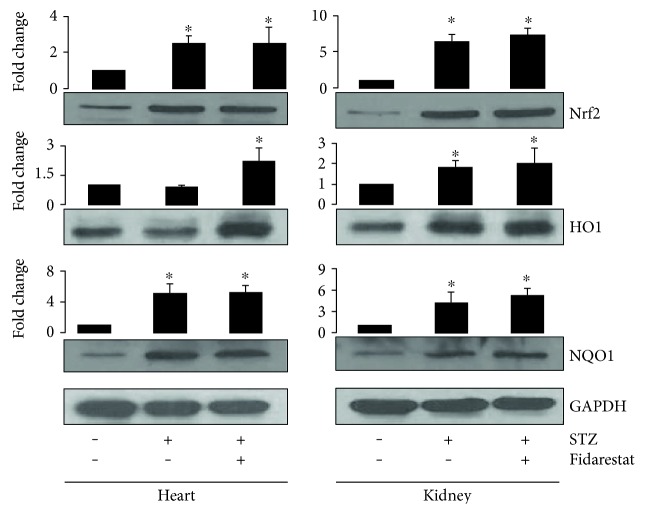
AR inhibitor induces the expression of Nrf2 and its dependent antioxidant enzymes in STZ-induced diabetic mice tissues. STZ-induced diabetic mice were treated without or with fidarestat as described in Section 2. An equal amount of proteins from the heart and kidney homogenates were subjected to Western blot analysis using specific antibodies against Nrf2, HO1, and NQO1. Fold changes were determined after normalizing with loading control, GAPDH. A representative blot from three independent analyses is shown. ^∗^*p* < 0.05 when compared with control, and #*p* < 0.05 when compared with the HG-treated group.

We next examined the effect of fidarestat on the expression of antioxidant proteins, HO1, and Nrf2 in STZ-induced diabetic mouse heart and kidney tissues. Similar to data shown in the Thp1 cells, AR-inhibitor (fidarestat) also augmented the STZ-induced increase in the expression of Nrf2, HO1, and NQO1 in the heart and kidney homogenates of mice (Figure 5).

### 3.6. AR Inhibitor Regulates HG-Induced Phosphorylation of AMPK-*α*1 in Thp1 Cells

**Figure 6:**
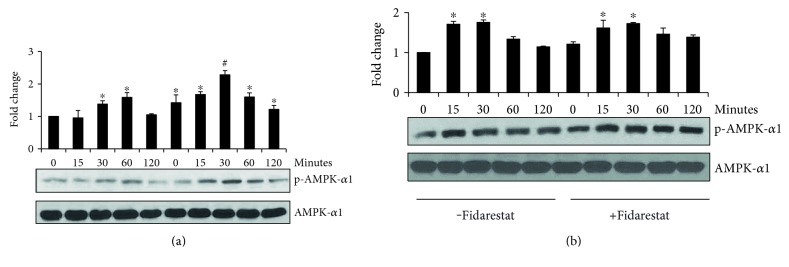
AR inhibition increases HG-induced phosphorylation of AMPK-*α*1 in Thp1 cells. (a) The Thp1 cells and (b) control and Nrf2-SiRNA-transfected cells were pretreated with fidarestat for overnight followed by incubation with HG for 15, 30, 60, and 120 min. Equal amounts of cytosolic proteins were subjected to Western blot analysis using antibodies against total and phospho-AMPK-*α*1. Fold changes were determined after normalizing with total AMPK-*α*1. A representative blot from three independent analyses is shown. ^∗^*p* < 0.05 when compared with control, and #*p* < 0.05 when compared with the HG-treated group.

Since AMPK-*α*1 activation has been shown to activate Nrf2 signals, we next investigated the effect of fidarestat on HG-induced AMPK-*α*1 activation in Thp1 cells. Our results shown in Figure 6(a) indicate that fidarestat increased the HG-induced phosphorylation of AMPK-*α*1 in Thp1 cells. Further, to investigate the effect of Nrf2 on AMPK-*α*1 activation, Nrf2-siRNA-transfected Thp1 cells were stimulated with HG ± fidarestat and examined AMPK-*α*1 activation. The results shown in Figure 6() indicate that HG-induced increase in the phosphorylation of Nrf2 in the absence of fidarestat. However, pretreatment of fidarestat to the Nrf2 knockdown cells did not show any significant differences in the phosphorylation of AMPK-*α*1. These results suggest that by regulating the Nrf2-mediated AMPK-*α*1, AR inhibitor could modulate hyperglycemic stress in Thp1 cells.

## 4. Discussion

We have shown previously that pretreatment with AR inhibitors prevents cytokine-, chemokine-, HG-, and LPS-induced inflammatory response mediated by NF-*κ*B in various cellular studies [12–15]. Further, AR inhibitors prevent NF-*κ*B-mediated proinflammatory pathways in vitro and in vivo models of hyperglycemia [12, 16]. However, it is not clear how pretreatment with AR inhibitors prepares cells against oxidative stress and activates Nrf2-mediated anti-inflammatory pathways. In this study, we have demonstrated that fidarestat induces Nrf2-mediated antioxidative and anti-inflammatory pathways in Thp1 monocytic cells. Further, fidarestat also augmented the HG-induced expression of Nrf2 and its downstream targets. These results suggest that preincubation with AR inhibitors prepares the cells to defend against pathological effects of hyperglycemia.

Nrf2 transcription factor regulates the expression of a number of cytoprotective antioxidative genes including *SOD*, catalase, *GSTs*, *AR*, *HO1*, *NQO1*, and so forth [9, 10]. Several studies indicate that antioxidants overexpress the Nrf2 pathway as a defense mechanism against various oxidative insults including hyperglycemia [17, 18]. Further, antioxidants such as flavonoids, triterpenoids, quinols, and tBHQ increase the activation of Nrf2 and protect against diabetes-induced nephropathy [19–23]. In addition, Nrf2 null mice are susceptible to the STZ-induced kidney injury [24]. Another study indicates that sulforaphane prevents metabolic dysfunction in hyperglycemia by increasing the expression of the Nrf2 pathway in human endothelial cells [25, 26]. Similarly, curcumin has been shown to decrease insulin resistance, improve pancreatic cell function, and reduce hyperglycemia-induced inflammatory response and complications by activating the Nrf2 pathway [27–29]. These studies suggest the significance of Nrf2 activation in diabetes complications. Consistent with these studies, our current data also suggest that treatment of Thp1 cells with fidarestat augmented the HG-induced Nrf2 activity indicating that fidarestat increases antioxidative balance in the cells and thereby regulates HG-induced cell viability. Furthermore, we have evaluated the effect of fidarestat on HG-induced Thp1 cell viability in Nrf2-ablated cells. Our results demonstrate that AR inhibitor prevented the HG-induced cell death in control cells but not in Nrf2-ablated cells suggesting that Nrf2-mediated antioxidative pathways are required for the actions of AR inhibitor.

Increased expression of Nrf2 leads to increased expression of enzymes linked to antioxidative (NQO1, GSTs, catalase, SOD) and anti-inflammatory (HO1, AR) functions that counteract against oxidative insults [10, 30]. HO1 is an anti-inflammatory protein, and its overexpression has been shown to prevent various inflammatory complications including diabetes [31]. Specifically, HO1 has been shown to protect against HG-induced retinal endothelial cells damage and also prevent vascular inflammatory response in hyperglycemia [32–34]. In this study, our results indicate that fidarestat increases the expression of HO1 and augments HG-induced HO1 in Thp1 cells as well as kidney and heart tissues of STZ-induced diabetic mice suggesting that anti-inflammatory activities of AR inhibition may be through activation of HO1. In addition, our studies also suggest that AR inhibitor increases the expression of SOD and catalase in Thp1 cells and HO1 and NQO1 proteins in STZ-induced diabetic mice heart and kidney tissues, indicating that AR inhibition prevents hyperglycemia-induced oxidative stress by overexpressing various antioxidative enzymes. Since activation of AMPK has been shown to be involved in the regulation of Nrf2 pathway [35], we have also examined the effect of AR inhibitor fidarestat on HG-induced changes in the phosphorylation of AMPK-*α*1 in Thp1 cells. Our results suggest that pretreatment with fidarestat stimulates phosphorylation of AMPK-*α*1 in HG-induced Thp1 cells. However, fidarestat pretreatment has no effect on the AMPK-*α*1 activation in Nrf2-ablated cells.

In conclusion, we have shown that AR inhibitor fidarestat prevents HG-induced Thp1 cell death by induction of Nrf2 expression; DNA binding activity; and expression of HO1, NQO1, SOD, and catalase via activation of AMPK-*α*1. This suggests that AR inhibition prevents hyperglycemia-induced complications by upregulating anti-inflammatory Nrf2 pathway in addition to downregulating the proinflammatory NF-*κ*B pathway.

## Abbreviations

AR: Aldose reductase
FBS: Fetal bovine serum
Fid: Fidarestat
HG: High glucose
HO1: Hemeoxygenase 1
NQO1: NAD(P)H-quinone dehydrogenase 1
Nrf2: Nuclear factor erythroid 2- (NF-E2-) related factor 2
MTT: 3-(4,5-Dimethylthiazol-2-Yl)-2,5-diphenyltetrazolium bromide
SOD: Superoxide dismutase
STZ: Streptozotocin.
